# Implementation of a dedicated 1.5 T MR scanner for radiotherapy treatment planning featuring a novel high-channel coil setup for brain imaging in treatment position

**DOI:** 10.1007/s00066-020-01703-y

**Published:** 2020-10-25

**Authors:** Veit Mengling, Christoph Bert, Rosalind Perrin, Siti Masitho, Thomas Weissmann, Sina Mansoorian, Hadi Siavooshhaghighi, Rolf Janka, Sylvain Doussin, Melanie Habatsch, Rainer Fietkau, Florian Putz

**Affiliations:** 1 Department of Radiation Oncology, Universitätsklinikum Erlangen, Friedrich-Alexander-Universität Erlangen-Nürnberg, Universitätsstraße 27, 91054 Erlangen, Germany; 2 Institute of Radiology, Universitätsklinikum Erlangen, Friedrich-Alexander-Universität Erlangen-Nürnberg, Maximiliansplatz 3, 91054 Erlangen, Germany; 3 Siemens Healthineers, Henkestraße 127, 91052 Erlangen, Germany

**Keywords:** MR-only treatment planning, Immobilization, Simulation, Stereotactic radiosurgery, Prostate

## Abstract

**Purpose:**

To share our experiences in implementing a dedicated magnetic resonance (MR) scanner for radiotherapy (RT) treatment planning using a novel coil setup for brain imaging in treatment position as well as to present developed core protocols with sequences specifically tuned for brain and prostate RT treatment planning.

**Materials and methods:**

Our novel setup consists of two large 18-channel flexible coils and a specifically designed wooden mask holder mounted on a flat tabletop overlay, which allows patients to be measured in treatment position with mask immobilization. The signal-to-noise ratio (SNR) of this setup was compared to the vendor-provided flexible coil RT setup and the standard setup for diagnostic radiology. The occurrence of motion artifacts was quantified. To develop magnetic resonance imaging (MRI) protocols, we formulated site- and disease-specific clinical objectives.

**Results:**

Our novel setup showed mean SNR of 163 ± 28 anteriorly, 104 ± 23 centrally, and 78 ± 14 posteriorly compared to 84 ± 8 and 102 ± 22 anteriorly, 68 ± 6 and 95 ± 20 centrally, and 56 ± 7 and 119 ± 23 posteriorly for the vendor-provided and diagnostic setup, respectively. All differences were significant (*p* > 0.05). Image quality of our novel setup was judged suitable for contouring by expert-based assessment. Motion artifacts were found in 8/60 patients in the diagnostic setup, whereas none were found for patients in the RT setup. Site-specific core protocols were designed to minimize distortions while optimizing tissue contrast and 3D resolution according to indication-specific objectives.

**Conclusion:**

We present a novel setup for high-quality imaging in treatment position that allows use of several immobilization systems enabling MR-only workflows, which could reduce unnecessary dose and registration inaccuracies.

**Electronic supplementary material:**

The online version of this article (10.1007/s00066-020-01703-y) contains supplementary material, which is available to authorized users.

## Introduction

Radiotherapy (RT) treatment planning is performed using computed tomography (CT) images to enable dose calculation based on individual patient anatomy. Magnetic resonance imaging (MRI), however, has become a requirement for optimal treatment planning [1–5], since it offers superior soft tissue contrast and detection of small contrast-enhancing lesions compared to CT [6–8]. Another advantage of MRI is the possibility to generate images with additional contrasts with techniques such as diffusion-weighted imaging [9, 10], perfusion-weighted imaging [11], or functional MRI with blood oxygen level-dependent contrast [12].

Optimal treatment planning frequently requires the same positioning for CT, MRI, and during radiotherapy treatment sessions. Standard MRI scanners are not generally set up for imaging in the RT treatment position, and, furthermore, radiofrequency coils used in diagnostic radiology do not allow patient immobilization in thermoplastic mask systems for brain and head and neck cancer irradiation.

Recently, MRI scanners with dedicated RT options and equipment became available from different vendors. These include RT-specific modifications like laser bridges, flat tabletops, larger bore sizes, and immobilization devices, as well as software algorithms to calculate pseudo-CTs based on MRI images to enable magnetic resonance(MR)-only workflows.

In this work, we share our initial experiences with the implementation of a dedicated MR scanner for RT treatment planning. We present ways to adapt the vendor-provided solutions to allow MR imaging with existing mask immobilization systems. This includes a novel receive coil setup for brain imaging, which is then compared to the vendor-provided setup. Furthermore, we describe the developed RT-optimized sequence protocols for treatment planning in prostate and stereotactic brain radiotherapy.

## Materials and methods

### Setup

A 1.5 T MAGNETOM Sola with the MAGNETOM RT Pro Edition (Siemens Healthineers, Erlangen, Germany) was installed in December 2018 and used for radiotherapy planning from March 2019. It has a bore size of 70 cm, second-order active shimming, and a maximal field of view (FOV) of 50 × 50 × 50 cm^3^. The maximum gradient amplitude is 45 mT/m and the maximum slew rate is 200 T/m/s.

It is equipped with the INSIGHT system (Qfix, Avondale, AZ, USA) including an MR-compatible flat tabletop with indexing capability. The flat tabletop allows imaging with the spine coil. The system also comes with coil holders (Qfix) for the 18-channel body coil and two four-channel flexible coils (Siemens Healthineers) that can be used to form a head coil. The body coil holder allows for reliable coil positioning without the coil touching the patient. An MR-compatible Lok-Bar (CIVCO Medical Solutions, Kalona, IA, USA) with three pins enables consistent positioning of immobilization devices.

Two laser systems are available for patient positioning. Aside from the standard internal MR laser, an additional MR-compatible external laser bridge (DORADOnova MR3T, LAP of America Laser Applications, Boynton Beach, FL, USA) was installed. It consists of six sagittal, transverse, and coronal lasers and allows patient localization, isocenter marking, and direct laser steering to set skin marks.

**Fig. 1:**
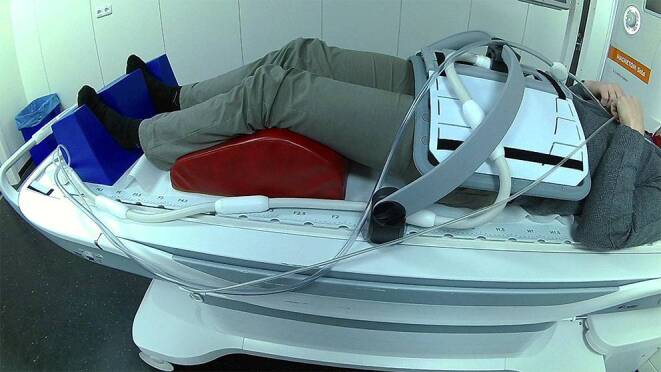
Setup for MR imaging in treatment position for patients with pelvic tumors. The *red *leg rest and the *blue* foot rest are identical to the ones used during radiotherapy treatment delivery. The coil holder in combination with the flat indexable tabletop allows for reproducible positioning

For prostate acquisitions, the same head and leg rest were used as in the planning CT scan in the RT treatment position. The receive coils used were the spine coil and the 18-channel Body Long coil suspended over the patient using a coil holder (see Fig. 1). The laser bridge is used for patient positioning as it allows for a more precise positioning due to the longer reach of the laser projection.

**Fig. 2:**
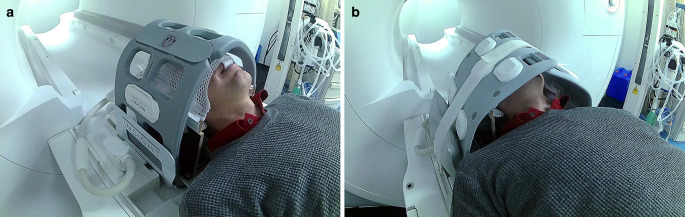
Setup for MR imaging in treatment position for brain tumor patients. **a** Vendor-provided setup with two four-channel Flex coils, **b** Our novel proposed setup with two 18-channel UltraFlex coils. In both setups, the coils are wrapped around the head of the patient, closing under the tabletop and above the nose of the patient. The most notable differences are the fixation and connection of the coils. The mask holder is a self-built wooden replica of the metallic one used during irradiation

**Fig. 3:**
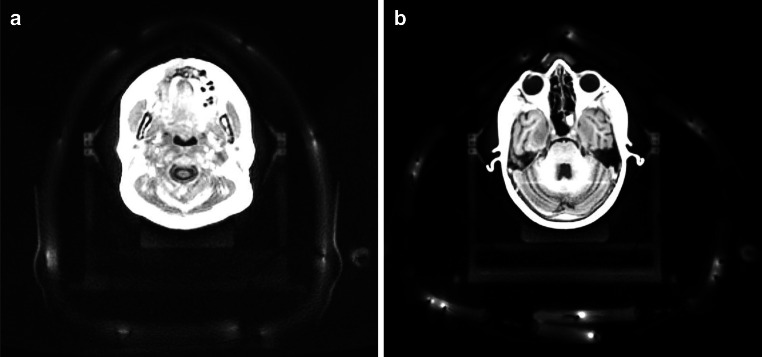
Axial view of **a** the four-channel Flex coil setup and **b** the 18-channel UltraFlex coil setup. As the image quality was worse in **a** and the coils have fewer visible elements, different windowing and slice positioning was chosen for the two images

For head acquisitions intended to guide stereotactic radiosurgery (SRS), patients were positioned with stereotactic mask immobilization (Brainlab, Munich, Germany). As the mask manufacturer did not provide MR-compatible mask holders at the time of implementation, an in-house built wooden mask holder was constructed that is compatible with the flat tabletop. Two surface coil setups with, respectively, 8 and 36 receiving channels were investigated (see Fig. 2 and 3): The vendor-suggested setup, which consists of two receiving coils (four-channel Flex Large) and the respective coil holder; and our novel setup, which also consists of two receiving coils (18-channel UltraFlex Large [Siemens Healthineers]) but significantly more receiving channels. The intention was to increase the signal-to-noise ratio (SNR) as well as image quality for target and OAR delineation, as the wooden mask holder induced an additional distance between the coils and the patient compared to the standard setup. As the UltraFlex coils are larger than the Flex coils, the coil holder could not be used in the novel setup. Reproducible positioning was instead achieved by placing cushions under the UltraFlex coils and fixing them with two Velcro straps at the top (see Fig. 2b).

### Image quality evaluation

As part of routine clinical practice, most patients received MR imaging in one of the above-described surface coil setups in treatment position as well as in a standard diagnostic setup (Head/Neck 20-channel coil) in the same session to enable optimal diagnostic assessment as well as dedicated imaging for treatment planning. To evaluate the image quality of the two setups in treatment position, the SNR of each setup was compared with the SNR in the standard diagnostic radiology setup (Head/Neck 20-channel coil). As the SNR was not homogeneous along the anterior–posterior direction, the evaluation was split into the anterior, the central, and the posterior part of the head. To calculate the SNR, the mean intensity in a circular region of interest (ROI) in white matter was divided by the standard deviation of a circular ROI in the background in the corresponding section. To ensure a homogeneous signal and that the coil profile does not affect SNR calculation, the circular ROIs were at least 0.5 cm^2^ in the white matter and between 4 and 5 cm^2^ in the background. The SNR was calculated on both the transverse T1w-MPRAGE sequence after contrast agent injection and the T2w-FLAIR (for detailed parameters see Tables 2 and 3).

The suitability for contouring was assessed by three experienced radiation oncologists (FP, TW, SM). Images of the three setups (154 in total) were blinded, loaded into 3DSlicer (v. 4.10.2) [13], and presented to the physicians in randomized order. The radiation oncologists then graded each image on a scale of 1 (not suitable for contouring) to 4 (excellent suitability for contouring), based on the image quality and the distinguishability of the lesions from the surrounding tissue using a custom-made software module in randomized order. Additionally, the radiation oncologists counted the number of metastases for every dataset with a similar software module in randomized order and in a blinded fashion.

The significance of the qualitative grading results was tested using a Welch two-sample t‑test. To compare the number of counted metastases between the diagnostic setup and the novel UltraFlex setup in treatment position, a paired t‑test was used. Calculations were performed using R and SPSS v.21 (IBM, Arnmonk, NY, USA) [14]. The level of significance was set at* p* > 0.05.

As the positioning in a thermoplastic mask was hypothesized to enable better immobilization than the standard diagnostic setup, motion artifacts in T1w-MPRAGE images of patients who received scans both in the treatment position and in the radiologic setup were compared. If motion artifacts were clearly identifiable in either OAR or target volumes, the image was qualified as motion corrupted.

### Sequence protocols

MRI sequences for radiotherapy planning should depict the three-dimensional boundaries of target volumes and organs at risk with the highest geometric accuracy and as clearly as possible. As the main emphasis of diagnostic imaging lies on the identification and characterization of diseases, dedicated sequence optimization for the purpose of RT planning is needed.

Whenever possible, isotropic 3D sequences should be used, as they reduce distortions and enable accurate multiplanar reconstructions [15]. Slice thickness should be as low as possible, with the exact value depending on site- and treatment-specific considerations and a general rule of thumb being that structures should be visualized on at least five slices to minimize over- or underestimation of volumes because of partial volume effects [15, 16]. As geometric precision is affected by various mechanisms in MR imaging, specific methods should be applied to counteract these effects. To decrease the geometric distortions caused by gradient nonlinearities, vendor-provided 3D distortion correction should always be applied as a minimum [15]. As susceptibility-induced distortions can lead to errors in frequency encoding direction, active shimming on a per patient basis should be used and the receiver bandwidth should be set as high as possible [15, 17, 18].

Before creating the core protocols, we formulated the following site- and disease-specific clinical objectives.

#### Brain metastases

MRI sequences in brain metastases should be able to depict the three-dimensional contrast-enhancing tumor volumes as accurately as possible without gaps. As brain metastases frequently measure 5 mm or less in diameter, resolution should be high in every image dimension to minimize partial volume effects. The contrast ratio between lesions and surrounding brain parenchyma should be optimized to allow accurate delineation and minimize inter-observer variability.

#### Gliomas

Similar considerations were applied for gliomas. However, emphasis on the most accurate depiction of the contrast enhancement was lower than in metastases, as the volume of contrast enhancement—if present—and clinically employed margins usually are much larger in gliomas than in metastases. In addition, the volume of contrast enhancement in malignant gliomas only represents a fraction of all tumor cells, with glioma cells extending far beyond the boundaries of the contrast-enhancing area. Therefore, more emphasis was put on accurate depiction of the surrounding T2w-FLAIR hyperintensity, which may represent non-enhancing tumor or microscopic disease extension. T2w-FLAIR hyperintensity should be depicted in high resolution and continuously without slice gaps.

As contrast-enhancing tumor and post-therapeutic changes are frequently difficult to differentiate in recurrent gliomas, additional information for contouring should be provided by a high-resolution diffusion-weighted sequence.

#### Prostate

A large volume including the inguinal lymph node levels and penile structures inferiorly and the common iliac lymph node levels superiorly should be imaged to allow for visualization of all pelvic lymph node levels.

The prostate, penile bulb, and pelvic lymph nodes should be visualized with good contrast to allow accurate delineation. In particular, it should be possible to clearly differentiate the lower boundary of the prostate, the urogenital diaphragm, and the penile bulb. In this regard, the main sequence should have a high resolution in all dimensions to allow for accurate delineation in both axial and sagittal reconstructions. An additional diffusion-weighted sequence with high resolution should be available to aid in visualization of macroscopic tumor inside the prostate.

## Results

### Patients

The data in this study result from patients who received an MRI scan within the first year after installation of the scanner. A total of 89 patients were included, with 19 patients receiving multiple scans. As the images were taken as part of standard clinical care, not all patients received imaging in a mask setup and the radiology setup. Additionally, not all patients received a T2w-FLAIR, as it was not needed for therapy in every case. In total, 11 T1w-MPRAGE images were acquired in the Flex coil setup, 83 in the UltraFlex coil setup, and 60 in the radiology setup. 10 T2w-FLAIR images were acquired in the Flex coil setup, 65 in the UltraFlex coil setup, and 37 in the radiology setup. For more details on the patients, see Supplementary Table 1.

**Table 1:** Mean signal-to-noise ratio (SNR) (± standard deviation) of the radiology setup (Head coil, *n* = 60), the vendor-provided setup (Flex coil, *n* = 83), and our novel setup (UltraFlex coil, *n* = 11). All coils are manufactured by Siemens Healthineers, Erlangen, Germany

	Head coil	Flex coil	UltraFlex coil
T1w MPRAGE			
Anterior	102 ± 22	84 ± 8	163 ± 28
Central	95 ± 20	68 ± 6	104 ± 23
Posterior	119 ± 23	56 ± 7	78 ± 14
T2w FLAIR			
Anterior	91 ± 11	62 ± 7	107 ± 13
Central	87 ± 9	58 ± 7	86 ± 11
Posterior	98 ± 16	51 ± 10	64 ± 8

### Motion artifacts

Of the 60 patients imaged in the diagnostic setup, 8 showed severe motion artifacts in the radiology setup that were visible in treatment-relevant regions. In contrast, no relevant motion artifacts could be detected in either the novel setup or the vendor-provided setup, in which patients were imaged in treatment position with mask immobilization.

### Signal-to-noise ratio

The mean SNR for the three setups is shown in Table 1. In the novel setup, it decreased from anterior to central and from central to posterior for both investigated sequences. The SNR in the vendor-provided setup also decreased from anterior to central and from central to posterior for both sequences. The anterior and central SNR in the radiology setup showed no significant difference, while the SNR in the posterior part of the head was higher than in the anterior and the central part of the head for both sequences.

**Fig. 4:**
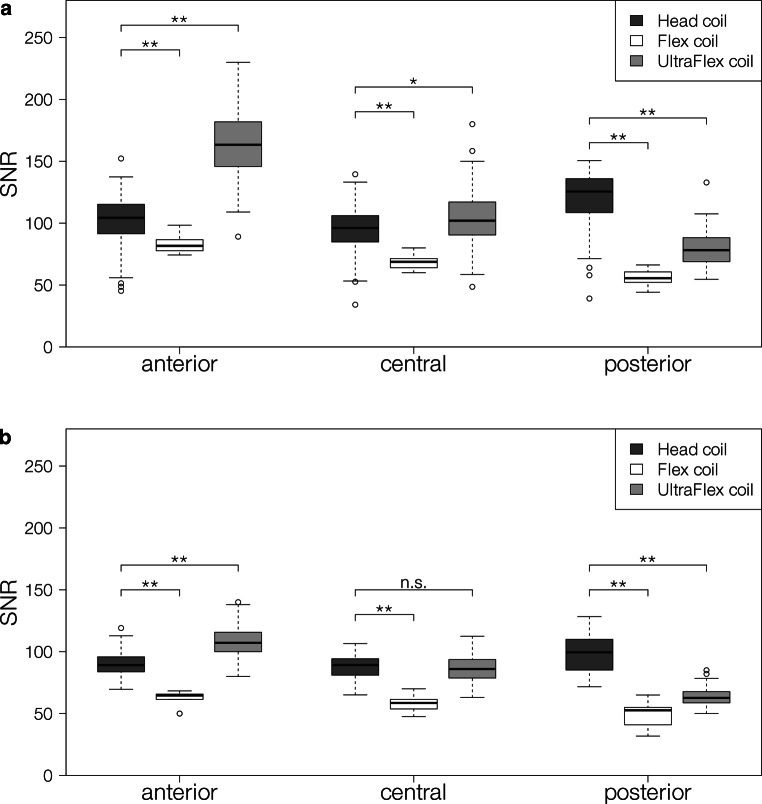
Boxplot of the signal-to-noise ratio (SNR) of the different coil setups in the anterior, central, and posterior part of the head measured on **a** the contrast-enhanced T1w-MPRAGE and **b** the T2w-FLAIR. * indicates a significance level of *p* < 0.05, ** indicates a significance level of *p* < 0.01. All coils are manufactured by Siemens Healthineers, Erlangen, Germany

The SNR of the novel setup for both sequences is higher than the SNR in the radiology setup anteriorly, but lower posteriorly. Centrally, it is higher than in the radiology setup for the T1w-MPRAGE. For the T2w-FLAIR, no significant difference was found centrally. In contrast, the SNR of the vendor-provided setup was lower anteriorly, centrally, and posteriorly. For more details of the distribution, see Fig. 4.

### Blinded expert-based assessment of image quality

**Fig. 5:**
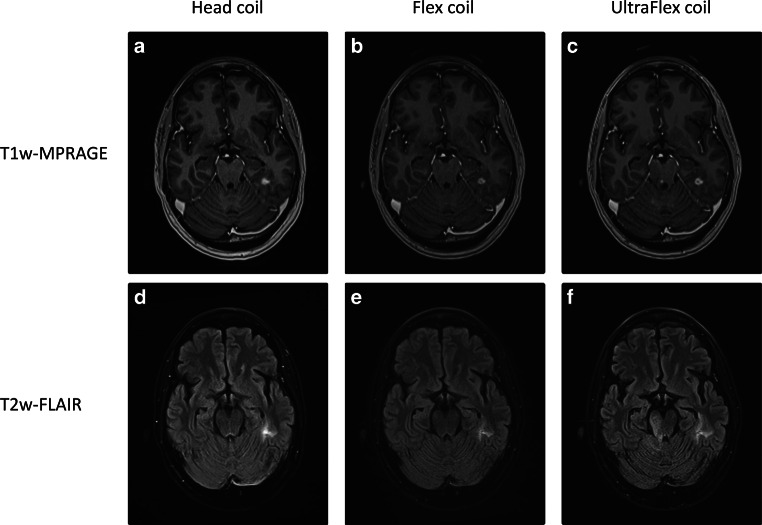
Images of a patient in all three investigated setups. **a–c** Images of the contrast-enhanced T1w-MPRAGE, **d–f **images of the T2w-FLAIR. **a,b,d,e** were acquired on the same day, **c,f** were acquired 77 days later. The contrast-enhancing lesion and the FLAIR-hyperintensity can be seen clearly in all three setups. While the Head coil shows relatively homogeneous signal-to-noise ratio, the noise in both mask setups increases significantly in anterior–posterior direction

The qualitative grading of the image quality showed a median score of 2 (“suitable for contouring”) for all three setups in a randomized and blinded assessment. No significant difference could be found between the setups (0.1 < *p* < 0.4). Furthermore, in a randomized and blinded comparison, there was no significant difference in the number of identified brain metastases between the diagnostic setup and the novel high-channel UltraFlex setup (mean number of identified brain metastases 3.4 vs. 3.2, *p* = 0.369). Fig. 5 shows images of a patient who received imaging in all three setups.

### Sequences used

According to the requirements and objectives defined in the previous section, the following core protocols were established. Detailed sequence parameters can be found in Tables 2 and 3. Brain measurements are always taken with the novel high-channel UltraFlex setup. All sequences use active shimming to reduce patient-induced distortions as well as 3D distortion correction to reduce system-induced distortions. To further reduce the effect of patient-induced distortions, the bandwidth was set to the highest value possible while keeping acceptable SNR and acquisition time.

**Table 2:** Detailed sequence parameters of the protocols used for head imaging

	T2w-TSE-FLAIR	T2w-SPACE-Dark-Fluid	T1w-MPRAGE	T1w-SPACE	EPI with shaped excitation (ZOOMit)
Voxel size (mm × mm × mm)	0.7 × 0.7 × 5.0	1.0 × 1.0 × 1.0	1.0 × 1.0 × 1.0	1.0 × 1.0 × 1.0	0.8 × 0.8 × 3.0
Orientation	Transverse	Sagittal	Transverse	Sagittal	Coronal
Dimension	2D	3D	3D	3D	2D
Contrast agent	Yes	Yes	Yes	Yes	Yes
Flip angle (°)	150	120 (T2 Var)	8	120 (T1 Var)	90
TR (ms)	9000	7600	2200	700	5000
TE (ms)	93	431	3.02	22	67
TI (ms)	2500	2400	900	–	–
Fat-saturation	Yes	Yes	No	Yes	Yes
Bandwidth (Hz/Px)	130	651	400	630	1447

**Table 3:** Detailed sequence parameters of the protocols used for prostate imaging

	T2w-SPACE	DIXON	T2w-BLADE	RESOLVE	EPI with shaped excitation (ZOOMit)
Voxel size (mm × mm × mm)	0.9 × 0.9 × 1.0	1.6 × 1.6 × 2.0	0.6 × 0.6 × 3.0	0.9 × 0.9 × 4.8	0.8 × 0.8 × 3.0
Orientation	Transverse	Transverse	Sagittal	Transverse	Transverse
Dimension	3D	3D	2D	2D	2D
Contrast agent	No	No	No	No	No
Flip angle (°)	170	15	160	180	90
TR (ms)	1200	6.2	5820	8060	4000
TE (ms)	122	2.39/4.77	117	60/98	70
Fat-saturation	No	No	No	Yes	Yes
Bandwidth (Hz/Px)	651	1120	178	994	1220

#### Brain metastases

Gadolinium-based contrast agent is injected immediately after completing the localizer. To ensure sufficient contrast uptake, a transverse T2w-FLAIR is acquired. After that, a high-resolution (1 × 1 × 1 mm^3^ isotropic) transverse T1w-MPRAGE is acquired, followed by a 1 × 1 × 1 mm^3^ isotropic sagittal T1w-SPACE. The total acquisition time was 17:40 min.

#### Gliomas

The core protocol also starts with contrast agent injection right after completing the localizer. A 1 × 1 × 1 mm^3^ isotropic sagittal T2w-DARK-FLUID is acquired next, followed by a 1 × 1 × 1 mm^3^ isotropic sagittal T1w-SPACE. For the diffusion sequence, an EPI-ZOOMIT with 0.8 × 0.8 ×3.0 mm^3^ resolution was chosen to enable a high-resolution assessment of diffusion-weighted image changes. The total acquisition time was 15:41 min.

#### Prostate

The core protocol for prostate acquisitions consists of a transverse T2w-SPACE with 0.9 × 0.9 × 1 mm^3^ resolution and compressed sensing acceleration with an acceleration factor of 8. The T2w-SPACE is the main sequence, with a FOV of 30 cm by 30 cm, and depicts the whole pelvis in high resolution. The T2w-SPACE is followed by a transverse DIXON with 1.6 × 1.6 × 2.0 mm^3^ and a sagittal T2w-BLADE with 0.6 × 0.6 × 3.0 mm^3^ resolution, which is used to additionally improve the delineation of the lower prostate boundary. Finally, a transverse EPI-ZOOMIT with 0.8 × 0.8 × 3.0 mm^3^ resolution and a transverse RESOLVE with a resolution of 0.9 × 0.9 × 4.8 mm^3^ are obtained to identify diffusion-restricted tumor foci. The acquired DIXON sequence is also used to generate a pseudo-CT using an atlas-based vendor-provided algorithm. The total acquisition time was 24:55 min.

## Discussion

In this work, we present our initial experiences with implementing a dedicated MRI for RT treatment planning. We introduced a novel receive coil setup as well as our core sequence protocols for brain stereotactic and prostate treatment planning.

Compared to the diagnostic gold standard, the mean SNR of the novel setup on both the T1w-MPRAGE and the T2w-FLAIR was better in the anterior part of the head, slightly better in the central part of the head (no significant difference for the T2w-FLAIR), and worse in the posterior part of the head. The variance of the SNR in the anterior–posterior direction of our novel setup was higher than in the radiology setup. The SNR in the novel setup was significantly higher in all parts of the head compared to the vendor-provided Flex coil setup. The anterior–posterior distribution was comparable. This can be explained by the distance of the receive coils to the imaged volume. In the radiology setup, the SNR is highest in the posterior part of the head, where the head lies directly over the receive element. The anterior and the central part of the head are further away from the coil, resulting in lower SNR. The same reasoning leads to the non-uniform distribution for the UltraFlex and Flex coil setup. While the anterior part of the head is in direct contact with the coil, the head rest and flat tabletop overlay led to a distance of about 9 cm between the receive coils and the back of the head. The lower SNR in the posterior part of the head might be improved by adding a small coil in the back of the mask holder. While the combination of coils would need to be tested, a superior SNR with our novel setup may likely be achievable in all parts of the head compared to the radiology setup in the Head/Neck 20 coil.

The median qualitative grading of the image quality showed no difference between the three tested setups. This means that the novel setup is not inferior to the diagnostic radiology gold standard, while allowing imaging in the treatment position. However, this also means that it is not significantly better than the setup with the smaller Flex coils. One possible explanation is the small sample size (*n* = 11) for the Flex coil setup compared the novel UltraFlex setup (*n* = 83). Another explanation is that the image quality of the two flexible coil setups highly depends on the inspected part of the head. This results in lower scores for posterior lesions compared to anterior lesions. The mean SNR in the posterior part of the head is also more similar between the setups than in the anterior part of the head, which could explain the similar ratings. Most images rated in this work had their lesions in the posterior part of the head. As no significant difference in the number of detected metastases was found between the setups, the SNR is still high enough to reliably detect the lesions. A limitation of the study was the bias introduced by the imaging time after contrast agent injection, since later contrast phases may also enhance the visibility of lesions [19]. When measurements were done with both a flexible coil setup and the radiological setup, the first sequence was always taken in the flexible coil setup. This may have resulted in a systematically better visibility of the lesion in the radiology setup because of the later contrast phase.

All coil setups produced images that were suitable for contouring. Our novel setup, however, combines the advantages of the vendor-provided setup and the standard radiology setup as it allows for high-quality imaging in RT treatment position. Additionally, positioning in a thermoplastic mask leads to reduced motion artifacts. In our case, no motion artifacts were observed in the mask setups, while some groups report movement to be less than 1.5 mm [20].

The current state-of-the art coil setup for RT treatment planning consists mainly of flexible surface loop coils with a low number of channels [21–23]. These have been reported to have a significantly worse SNR than diagnostic coils [22]. In comparison, the SNR of our novel setup is significantly higher both anteriorly and centrally, and lower posteriorly compared to the diagnostic coil setup, while having the same suitability for contouring. Therefore, our novel setup can be seen as an improvement over the state-of-the-art setup. The setup is also less prone to setup errors, as the coils only fit under the tabletop in a specific way. This reduces the influence of technician-dependent coil positioning, which can be a problem for surface flexible loop coils [24].

Another advantage of our setup is the possibility to use almost all mask immobilization systems, which usually do not fit into the diagnostic head coil. For example, our setup can be extended for head and neck examinations by adding the Body Long coil with the body coil holder to cover the neck area. Different mask systems will most likely need adapters to attach the masks to the tabletop. If not commercially available, these can be built relatively easily, as demonstrated by our in-house built wooden mask holder.

The protocols we developed are in accordance with a recently published consensus paper on MRI simulation [15]. Active shimming, 3D acquisition, and distortion correction were applied whenever possible.

In our brain metastases protocol, we acquire two high-resolution, contrast-enhanced T1w images. Currently, the T1w-MPRAGE is still the most widely used sequence for imaging of brain tumors [25, 26]. However, there is growing evidence that the T1w-SPACE could be superior to the T1w-MPRAGE for intracranial target volume delineation [25, 27, 28]. Therefore, we performed both sequences for brain metastases. While we could see the target volumes better on the T1w-SPACE in most cases, the T1w-MPRAGE still sometimes provided better contrast. The generally superior conspicuity of lesions in the T1w-SPACE in our experience can be largely attributed to the lower contrast between white matter and grey matter. It is often beneficial to acquire diffusion-weighted images of the whole brain to aid tumor visualization, especially if treatment-related contrast enhancement is present following surgery or radiation. This can be achieved by an EPI sequence. For a limited field-of-view an additional EPI-ZOOMIT can be useful, which provides better resolution.

The glioma protocol features a high-resolution T2w-DARK-FLUID that allows high-resolution imaging of the T2w-FLAIR hyperintensity. As this sequence is significantly longer than a standard T2w-FLAIR, we decided to only include one contrast-enhanced T1w sequence. We chose the T1w-SPACE because it has been shown to provide favorable conspicuity and contrast ratio in comparison to the T1w-MPRAGE in patients with gliomas [25]. The EPI-ZOOMIT allows high-resolution diffusion imaging of the investigated volume.

In our prostate protocol, we chose to use a transverse T2w-SPACE instead of a commonly recommended T2w-TSE [15]. This allows the whole pelvis to be imaged in a high-resolution 3D-volume. The T2w-BLADE has the added benefit of reducing motion artifacts caused by breathing or uncooperative patients. The diffusion-weighted sequences, especially the EPI-ZOOMIT, provide high-resolution diffusion imaging of the prostate.

Our experiences in fine-tuning the protocols from the starting point of the diagnostic sequence settings showed that standard parameters like TE, TR, or TI are almost always adjusted optimally for radiotherapy uses, too. The voxel resolution in diagnostic sequences, however, can be slightly non-isotropic, which is undesirable for RT purposes. Additional standard diagnostic sequences often only employ 2D-distortion correction, if any, which is potentially a relic from times in which 3D-distortion correction was computationally relatively demanding. Although most sequences come with active shimming enabled by default, the shimming type should be checked for every sequence. As we only changed parameters that could diminish the quality of the imaging and the sequences, we optimized for precision based on diagnostic sequences; the resulting precision should be at least equal to that of the well-established diagnostic sequences. Protocol time is also a major factor in protocol development. Therefore, it is important to get a feeling for which acceleration techniques yield the optimal results for each sequence type. While most sequences in our protocols are accelerated by parallel imaging techniques such as GRAPPA [29], we also tested compressed sensing. The success of this technique highly depends on the sequence it is used on. For the T2w-SPACE used in the prostate protocol, an acceleration factor of 8 with compressed sensing produced high-quality images with significantly reduced acquisition time. Compressed sensing resulted in blurred contours of the metastases when applied to the T1w-SPACE in our brain protocols, making it unusable with our current parameters. While the higher coil number of the UltraFlex setup would theoretically allow for higher acceleration factors in parallel imaging, our experiences showed that the loss of SNR with higher acceleration factors, especially posterior, is not tolerable.

The setup presented in this work is optimally suited for an MR-only workflow, where imaging has to be performed in treatment position. The diagnostic image quality that can be achieved with our setup, as well as the possibility to generate head pseudo-CTs based on these images for dose calculation [30–33], make it an optimal choice for MR-only workflows in head treatments. Head-Neck MR-only workflows may be realized by adding the body coil to our setup to produce high-quality head and neck images. A prostate MR-only workflow can also be realized by calculating a pseudo-CT based on the MRI images. This leads to a more efficient workflow, reducing the number of examinations for a patient and therefore avoiding additional ionizing radiation [34]. Vendor-provided automated algorithms to calculate pseudo-CTs are available on our MR scanner that enable MR-only workflows without the need for additional planning CTs and image coregistration [35]. We are currently evaluating the dosimetric accuracy of the pseudo-CTs. While in our experience problems may arise in postoperative situations, other groups have found excellent agreement for focal brain VMAT radiotherapy with D_95%_ differences of 0.0% [36].

## Conclusion

In this work, we presented a novel setup for brain imaging in treatment position with mask immobilization. We showed that with two UltraFlex coils, diagnostic image quality in treatment position with mask immobilization can be achieved. By building a mask holder that fits the specific immobilization system, our coil setup could be used for a range of mask systems. We also shared our initial experiences with implementing dedicated RT planning protocols and presented the core protocols we employed for radiotherapy treatment planning.

## Caption Electronic Supplementary Material

Supplementary Table 1: Patient statistics for all patients included in this work.
