# Supplementary material for: Implementation of a dedicated 1.5 T MR scanner for radiotherapy treatment planning featuring a novel high-channel coil setup for brain imaging in treatment position
Source: Strahlenther Onkol. 2020 Oct 25;197(3):246–56. doi: 10.1007/s00066-020-01703-y (PMC7892740; doi:10.1007/s00066-020-01703-y)
Supplement: Supplementary file 1 — Supplementary Table 1: Patient statistics for all patients included in this work. [file 66_2020_1703_MOESM1_ESM.docx]

**Supplementary Table 1:** Patient statistics for all patients included in this work.

| **Patient ID** | **Age** | **Sex** | **Diagnosis** |
| --- | --- | --- | --- |
| P01 | 46 | m | Metastases |
| P02 | 59 | m | Metastases |
| P03 | 57 | m | Metastases |
| P04 | 52 | f | Metastases |
| P05 | 56 | m | Astrocytoma WHO grade 2 |
| P06 | 54 | m | Metastases |
| P07 | 51 | f | Pituitary adenoma |
| P08 | 66 | f | Astrocytoma WHO grade 4 |
| P09 | 40 | f | Metastases |
| P10 | 71 | f | Metastases |
| P11 | 54 | m | Metastases |
| P12 | 64 | f | Metastases |
| P13 | 61 | f | Metastases |
| P14 | 60 | f | Metastases |
| P15 | 64 | f | Metastases |
| P16 | 59 | m | Metastases |
| P17 | 39 | m | Metastases |
| P18 | 69 | m | Metastases |
| P19 | 79 | f | Metastases |
| P20 | 70 | f | Metastases |
| P21 | 71 | f | Metastases |
| P22 | 72 | m | Metastases |
| P23 | 64 | m | Metastases |
| P24 | 18 | m | Astrocytoma WHO grade 4 |
| P25 | 51 | f | Astrocytoma WHO grade 3 |
| P26 | 60 | f | Astrocytoma WHO grade 4 |
| P27 | 65 | m | Metastases |
| P28 | 59 | f | Metastases |
| P29 | 55 | m | Metastases |
| P30 | 77 | m | Metastases |
| P31 | 70 | m | Metastases |
| P32 | 19 | m | Astrocytoma WHO grade 1 |
| P33 | 56 | m | Astrocytoma WHO grade 3 |
| P34 | 63 | f | Metastases |
| P35 | 67 | f | Metastases |
| P36 | 34 | m | Metastases |
| P37 | 68 | m | Metastases |
| P38 | 47 | f | Metastases |
| P39 | 64 | m | Pituitary adenoma |
| P40 | 59 | f | Astrocytoma WHO grade 4 |
| P41 | 68 | f | Metastases |
| P42 | 45 | f | Astrocytoma WHO grade 4 |
| P43 | 60 | f | Metastases |
| P44 | 54 | f | Metastases |
| P45 | 63 | f | Metastases |
| P46 | 69 | m | Metastases |
| P47 | 61 | m | Astrocytoma WHO grade 4 |
| P48 | 58 | f | Astrocytoma WHO grade 4 |
| P49 | 76 | f | Astrocytoma WHO grade 3 |
| P50 | 42 | f | Pituitary adenoma |
| P51 | 36 | m | Astrocytoma WHO grade 4 |
| P52 | 63 | m | Metastases |
| P53 | 59 | m | Metastases |
| P54 | 77 | f | Metastases |
| P55 | 82 | f | Acoustic neuroma |
| P56 | 60 | m | Metastases |
| P57 | 50 | m | Astrocytoma WHO grade 4 |
| P58 | 59 | m | Metastases |
| P59 | 62 | f | Metastases |
| P60 | 56 | f | Metastases |
| P61 | 82 | m | Metastases |
| P62 | 51 | m | Astrocytoma WHO grade 4 |
| P63 | 79 | m | Astrocytoma WHO grade 4 |
| P64 | 63 | f | Metastases |
| P65 | 54 | f | Metastases |
| P66 | 61 | m | Metastases |
| P67 | 64 | m | Metastases |
| P68 | 73 | f | Metastases |
| P69 | 51 | m | Astrocytoma WHO grade 3 |
| P70 | 70 | f | Metastases |
| P71 | 63 | m | Metastases |
| P72 | 73 | f | Metastases |
| P73 | 38 | f | Metastases |
| P74 | 47 | f | Metastases |
| P75 | 54 | m | Metastases |
| P76 | 66 | f | Metastases |
| P77 | 57 | m | Metastases |
| P78 | 58 | m | Metastases |
| P79 | 57 | m | Metastases |
| P80 | 57 | m | Astrocytoma WHO grade 4 |
| P81 | 72 | f | Metastases |
| P82 | 56 | m | Metastases |
| P83 | 81 | m | Metastases |
| P84 | 52 | f | Astrocytoma WHO grade 4 |
| P85 | 74 | m | Metastases |
| P86 | 65 | m | Metastases |
| P87 | 72 | m | Metastases |
| P88 | 73 | f | Metastases |
| P89 | 66 | f | Metastases |
